# Acute Cervical Longitudinally Extensive Transverse Myelitis in a Child With Lipopolysaccharide-Responsive-Beige-Like-Anchor-Protein (LRBA) Deficiency: A New Complication of a Rare Disease

**DOI:** 10.3389/fped.2020.580963

**Published:** 2020-10-16

**Authors:** Matteo Chinello, Margherita Mauro, Gaetano Cantalupo, Giacomo Talenti, Sara Mariotto, Rita Balter, Massimiliano De Bortoli, Virginia Vitale, Ada Zaccaron, Elisa Bonetti, Daniela Di Carlo, Federica Barzaghi, Simone Cesaro

**Affiliations:** ^1^Pediatric Hematology Oncology, Azienda Ospedaliera Universitaria Integrata, Verona, Italy; ^2^Pediatric Department, Santa Maria Degli Angeli Hospital, Pordenone, Italy; ^3^Child Neuropsychiatry, University of Verona, Verona, Italy; ^4^Department of Diagnostics and Pathology, Neuroradiology Unit, Verona University Hospital, Verona, Italy; ^5^Neurology Unit, Department of Neurosciences, Biomedicine and Movement Sciences, University of Verona, Verona, Italy; ^6^Pediatric Department, University of Verona, Verona, Italy; ^7^Pediatric Immunohematology and Bone Marrow Transplantation Unit, San Raffaele Telethon Institute for Gene Therapy, Milan, Italy

**Keywords:** lipopolysaccharide responsive beige-like anchor protein (LRBA), myelitis, acute cervical longitudinally extensive transverse myelitis, common variable immune deficiency (CVID), autommunity

## Abstract

Lipopolysaccharide responsive beige-like anchor protein (LRBA) deficiency is a primary immunodeficiency disorder (PID) that can cause a common variable immunodeficiency (CVID)-like disease. The typical features of the disease are autoimmunity, chronic diarrhea, and hypogammaglobulinemia. Neurological complications are also reported in patients affected by LRBA deficiency. We describe a 7-year old female with an acute cervical longitudinally extensive transverse myelitis (LETM) as a feature of LRBA deficiency. This is the first case of LETM associated with LRBA deficiency described in literature.

## Introduction

Lipopolysaccharide responsive beige-like anchor protein (LRBA) deficiency is a primary immunodeficiency disorder (PID) described as a cause of common variable immunodeficiency (CVID)-like disease (1). Several genes responsible of different subgroups of CVID have been identified (2), but the majority of patients with CVID have an unknown genetic etiology. CVID is a diagnosis of exclusion and so it is not surprising that CVID has heterogeneous clinical and laboratory presentations (3). This disease can be caused by LRBA gene defects (1). LRBA is a member of BEACH-WD40 protein family and it is expressed in several tissues (1, 4). The LRBA gene is located on 4q31.3, contains 57 exons and encodes a protein containing 2851 amino acid residues (5). The LRBA protein is widely expressed in several cell types including hematopoietic, neural, gastrointestinal, and endocrine cells^1^ with an high expression especially in lymphocytes (1, 6). This intracellular protein regulates the lysosomal degradation of cytotoxic T lymphocytes antigen-4 (CTLA-4), an inhibitory checkpoint receptor on T cells (6). For this reason patients with LRBA deficiency present an increase in CTLA4 degradation with clinical signs similar to CTLA4 haploinsufficient individuals (7, 8). An increase expression of LRBA is also described in many cancers, suggesting that the protein promotes cell survival by inhibiting apoptosis (9). The clinical features in patients affected by LRBA deficiency are heterogeneous with age of presentation ranging from 2 months to 12 years. There is not a genotype-phenotype correlation (10). LRBA deficiency can present with a wide spectrum of clinical manifestations such as inflammatory bowel disease (IBD)-like enteropathy, splenomegaly, pneumonia, autoimmune disease (AID) like immune thrombocytopenia purpura (ITP) and autoimmune hemolytic anemia (AIHA), hypogammaglobulinemia, B-cell deficiency, reduction in numbers of CD4+ T cells and regulatory T cells and autophagy (1, 6, 10–13). LRBA deficiency is suspected on the basis of heterogeneous clinical manifestations and immunological dysregulation that can be found through blood tests. The diagnosis currently relies on gene sequencing approaches or on the detection of LRBA protein by flow cytometry (14). The conventional treatment options for this disease have included various immunosuppressive agents such as corticosteroids, sirolimus, abatacept (soluble CTLA4- immunoglobulin fusion protein) (6, 13, 15) or Hematopoietic Stem Cell Transplantation (HSCT) (16). Neurological complications in patient affected by LRBA deficiency are described (6, 10, 17) even if they are not one of the typical features of the disease. We describe a 7-year old female with an acute cervical longitudinally extensive transverse myelitis (LETM) as a feature of LRBA deficiency. This is the first case of LETM associated with LRBA deficiency described in literature.

## Case Report

A 7-year-old female affected by LRBA deficiency was referred to our hospital for fever, stiff neck, and right cervical lymphadenopathy, that were unresponsive to anti-inflammatory drugs and oral antibiotic therapy.

The patient was born to unrelated parents after a full-term gestation. The birth weight was 2,420 g. The girl had normal psychomotor development. From 6 months of age she began to present recurrent severe infections during neutropenia associated with diffuse lymphadenomegaly (inguinal, abscellar, laterocervical, and mesenteric) and a diagnosis of autoimmune neutropenia was made. At 18 months she was hospitalized for cholelithiasis and hepatomegaly with autoimmune hepatitis and initial signs of cirrhosis. At 2 years she developed chronic diarrhea, hypertriglyceridemia, diffuse lipodystrophy, and splenomegaly. Double negative T cells resulted high (2.6% of total lymphocytes, normal value NV < 1.7%) and FAS-mediated apoptosis was abnormal. Genetic tests for autoimmune lymphoproliferative syndrome (ALPS) were negative (FAS, FAS ligand and caspase 10). In the suspicion of (ALPS)-like phenotype disease a therapy with mycophenolate mofetil was started. After one year, for the worsening of the lipodystrophy, anakinra, and then canakinumab were administered with initial but temporary clinical improvement. For persistent hypogammaglobulinemia the patient needed monthly administrations of immunoglobulins. At 4.5 years she underwent splenectomy for splenic abscess and started prophylaxis with amoxicillin/clavulanate and antiplatelet therapy with aspirin. At 5 years a mutation of Insulin Receptor Substrate 1 (IRS-1) which could lead to insulin resistance, leptin deficiency, and steatohepatitis, was detected. Therefore, leptin was administered with reduction of liver dimension, improvement of triglycerides level and no longer needing of insulin. The patient started empirical immunosuppressive therapy with prednisone (max 1 mg/kg/day) and sirolimus due to poor response to previous therapy. After few months the LRBA deficiency was identified by next generation sequencing (NGS) demonstrating double heterozygosity for two LRBA mutations; one inherited from the father (non sense c.7681 C>T: p.Q2561X) and one from the mother (splice disruption, c.1359 + 1G>A). Only sirolimus therapy was ongoing at the time of admission to our department.

**Figure 1:**
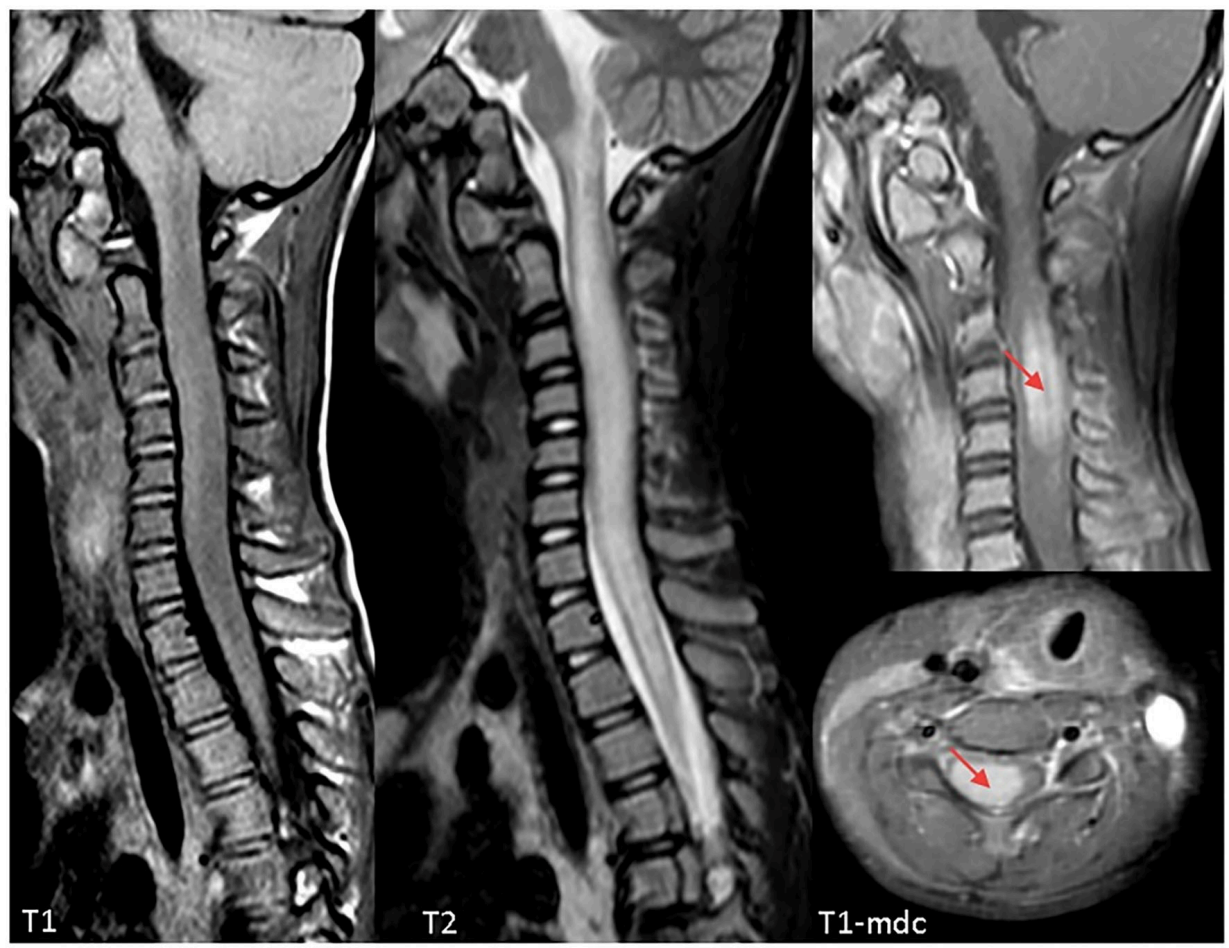
Cervical Magnetic Resonance Imaging (MRI) at clinical onset demonstrating a longitudinally extensive signal abnormality involving the cervical spinal cord, extending from the obex to D3. The cord appears markedly swollen, both gray and white matter are involved, and post-contrast sequences demonstrate marked cord enhancement between C3 and C5 (red arrows). Findings are compatible with acute longitudinally extensive transverse myelitis (LETM).

**Figure 2:**
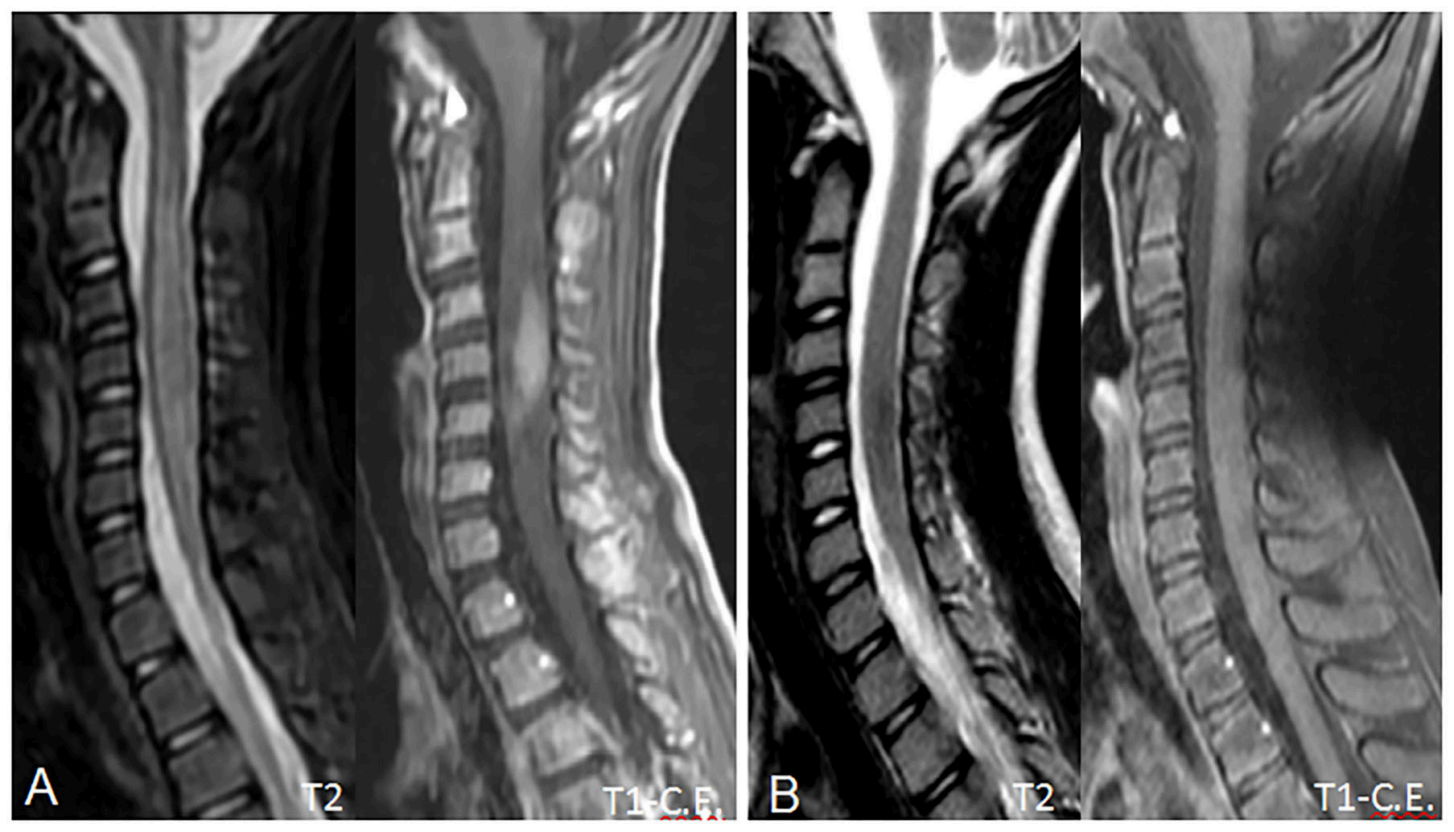
Follow-up MRI scans at 20 **(A)** and 55 **(B)** days, showing progressive reduction of the spinal cord swelling and contrast enhancement. In **(B)**, contrast enhancement has completely regressed, due to blood-brain barrier restoration, and the spinal cord shows a near-normal appearance with no residual disease. No spinal cord atrophy is noted.

On admission, she was conscious alert and oriented. Her heart rate, respiratory rate, blood pressure and body temperature were within normal range. She presented severe cervical pain associated with left deviation of the neck. The low-grade fever (37.6–37.8°C) and the stiff neck appeared 10 days earlier, but the pain was initially moderate and the patient was treated with anti-inflammatory drugs and oral antibiotic therapy. She received the last administrations of immunoglobulins 2 days before hospitalization. On examination, a right laterocervical lymphadenomegaly was observed. After few hours she developed right upper limb hyposthenia and fecal incontinence. Blood exams showed an increase of white blood cells (WBC) with low inflammatory markers (Table 1). Microbiological analysis were negative (blood cultures, urine culture, stool culture; Cytomegalovirus (CMV), Epstein Barr Virus (EBV), Adenovirus and Toxoplasma gondii serology; antistreptolysin antibodies, EBV, and CMV DNA copy number quantification by real-time polymerase chain reaction). Magnetic Resonance Imaging (MRI) was performed and showed extensive spinal cord T2- hyperintense lesion extending from the medulla oblongata to D3 level, with significant spinal cord swelling and marked contrast enhancement at C3–C5 level which was compatible with acute Longitudinally Extensive Transverse Myelitis (LETM) (Figure 1). Cerebrospinal fluid (CSF) analysis revealed an increase of white blood cells (100% lymphocytes), proteins and IgG levels with high CSF/serum albumin quotient and normal IgG-index (Table 2). CSF analysis for bacterial and viral infections yielded negative results (*Escherichia coli*, Haemophilus influenzae, Listeria monocytogenes, Neisseria meningitidis, Streptococcus agalatiae, Streptococcus pneumoniae, EBV, CMV, Herpes simplex Virus 1 (HSV1), Herpes simplex Virus 2 (HSV2), Human parechovirus, Varicella zoster virus (VZV), Enterovirus and Cryptococcus neoformans). Serum antibodies to myelin oligodendrocyte glycoprotein (MOG live cell-based assay) and aquaporin-4 (AQP4 fixed cell-based assay, Euroimmune commercial kit-4) were negative. Isoelectrofocusing revealed the presence of oligoclonal bands in both serum and CSF type 4 “mirror” pattern. An extensive autoimmune screening was negative [antinuclear antibodies (ANA), anti-double stranded DNA antibodies (anti-ds-DNA), anti-neutrophil cytoplasmic antibodies (ANCA, p-ANCA, c-ANCA, a-ANCA, anti PR3, anti myeloperoxidase antibodies MPO), anti-saccharomyces cerevisiae antibodies (ASCA), anti-smooth muscle antibody (ASMA), anti-liver-kidney microsome antibodies (LKM), anti-insulin antibody, TSH receptor antibodies (TRAb)]. The patient received intravenous dexamethasone at the dosage of 0.15 mg/kg for 27 days followed by a 9-week tapering dose of oral steroids which resulted in a rapid clinical improvement. Follow-up MRIs, performed after 20 and 55 days, respectively, (Figure 2), showed progressive improvement of spinal cord oedema and enhancement in parallel with a complete clinical recovery. The patient was then transferred to another institution for stem cell transplantation and the last follow-up MRI before transplant she was referred completely negative (image not available).

**Table 1:** Blood exams.

	**Value**	**Normal value**
White blood cells (WBC)	20.840/mm^3^	4,500–13,500
Neutrophils	13.970/mm^3^	600–6,400
C-reactive protein (CRP)	6 mg/L	<5

**Table 2:** Cerebrospinal fluid (CSF) analysis.

	**Value**	**Normal value**
Cellularity	70 cells/uL (100% lymphocytes)	<8
Glucose level	50 mg/dL	50–81
Protein content	1.35 g/L	0.15–0.45
IgG levels	82 mg/L	<34 mg/L
Albumin	874 mg/L	<320 mg/L
CSF/serum albumin quotient	23.75	<7
IgG-index	0.46	<0.70

## Discussion

Patients with LRBA deficiency typically present immunodeficiency, inflammatory bowel disease (IBD)-like enteropathy, and autoimmune disease (AID) (1, 11–13). Some studies report an imbalance in Th subsets, in particular in Th1-like Th17 and Treg cells and their corresponding cytokines in LRBA deficiency. This condition might be important in the immunopathogenesis of autoimmunity and enteropathy (18). According to some publications a defect of LRBA can be associated with an IPEX-like (19) or ALPS-like (20) clinical presentation thus similar to our patient's phenotype. Neurological complications of the disease have been described (6, 10, 17). Alkhairy et al. (10) reported that neurological features occur in 23% of patients. The authors, with the exception of myasthenia gravis which has an autoimmune etiology, describe patients with cerebral lesions and nervous tissue atrophy. They report two patients with cerebral granulomas, one with granuloma-like lesion with demyelination resulting in unilateral optic nerve atrophy, one with unilateral optic nerve atrophy, one with cerebral and cerebellar atrophy and another with parietal lobe lesion complicated by seizures (10). Tesi et al. described a patient with LRBA deficiency complicated with an acute disseminated encephalomyelitis (ADEM) who underwent hematopoietic stem cell transplant (17). ADEM has also been described in one CTLA-4 haploinsufficient patient (7) and in patient with CVID (21). To the best of our knowledge this is the first ever reported patient with LETM associated with LRBA deficiency. We believe that the high value of white blood count is a consequence of inflammation. The exclusion of infectious and neoplastic nature of the lesion, together with the good response to steroid treatment, allow us to consider it an inflammatory seronegative LETM. This condition is observed in a group of inflammatory autoimmune disorders, often in the context of neuromyelitis optica spectrum disorders (NMOSD) (22–25). These disorders are mostly related to the presence of antibodies against aquaporin (AQP4) or against myelin oligodendrocyte glycoprotein (MOG) (24–26). However, in some patients AQP4-Antibodies and MOG-Antibodies are lacking, thus these patients are usually labeled “double seronegative LETM” or “seronegative LETM” (26, 27). We consider LETM as one of the possible immune-mediated manifestations that compose the clinical spectrum of LRBA deficiency.

## Data Availability Statement

The original contributions presented in the study are included in the article/supplementary material, further inquiries can be directed to the corresponding author/s.

## Author Contributions

MC: involvement in medical diagnosis and follow up of the patient, first writer of the manuscript. MM: involvement in medical diagnosis of the patient and she helped to write the manuscript. GC, GT, SM, RB, MD, VV, AZ, EB, and DD: involvement in diagnosis and management of the patient. FB involvement in genetic diagnosis. SC: involvement in diagnosis and management of the patient, supervision of the process of the manuscript. All authors read and approved the final manuscript.

## Conflict of Interest

The authors declare that the research was conducted in the absence of any commercial or financial relationships that could be construed as a potential conflict of interest.

## Footnotes

^1^GeneCards. http://www.genecards.org/cgi-bin/carddisp.pl?gene=LRBA
